# EXAMINING THE IMPACT OF LONG-TERM CARE INSURANCE ON LABOR MARKET PARTICIPATION OF INFORMAL CARERS IN CHINA

**DOI:** 10.1093/geroni/igae098.0462

**Published:** 2024-12-31

**Authors:** Wei Yang, Xingtong Pei, Mingming Xu

**Affiliations:** King’s College London, London, England, United Kingdom; Sun Yat-sen University, Guangzhou, Guangdong, China (People’s Republic); Sun Yat-sen University, Guangzhou, Guangdong, China (People’s Republic)

## Abstract

Existing evidence from high-income countries suggests that long-term care insurance (LTCI) aimed at enhancing access to formal care can reduce the burden on informal carers and facilitate their reentry into the labor market. However, there is limited evidence regarding the specific carers who have been most impacted by such insurance. Drawing data from the China Health and Retirement Longitudinal Study of 2011, 2013, 2015, and 2018, we employ a staggered difference-in-differences model with propensity score matching to analyze the impact of LTCI on care burden reduction and labor market participation of the informal carers. Our study shows that LTCI substantially alleviates the burden on informal carers and promotes their labor market participation. Specifically, LTCI reduced care burden among spouses by 8.5 hours per month. We also found that LTCI’s impact on enhancing labor market participation was more significant among younger household members, reflected in an average income increase of 4,534 yuan per year. Our subgroup analysis highlights that LTCI primarily benefits informal carers providing care for older people with low income or those who were farmers or previously engaged in informal sectors. We urge the government extend the insurance program to additional regions and broaden its scope to include individuals from lower socioeconomic backgrounds.

